# Communicating with medical library users during COVID-19

**DOI:** 10.5195/jmla.2021.1003

**Published:** 2021-01-01

**Authors:** Dana Haugh

**Affiliations:** 1 dana.haugh@yale.edu, Web Services Librarian, Harvey Cushing/John Hay Whitney Medical Library, Yale University, New Haven, CT

## Abstract

**Background:**

The Harvey Cushing/John Hay Whitney Medical Library serves a community of over 22,000 individuals primarily from the Yale Schools of Medicine, Public Health, and Nursing and the Yale New Haven Hospital. Though they are geographically close to one another, reaching these disparate populations can be a challenge. Having a clear and thorough communication plan has proved invaluable in transcending communication chasms, especially in recent times of crisis.

**Case Presentation:**

This article describes the Harvey Cushing/John Hay Whitney Medical Library's methods for communicating and promoting its remote resources and services in response to coronavirus disease 2019 (COVID-19). It details our communication strategies and messages leading up to, and after, the Yale campus was closed and specifies how we pivoted from reaching users inside the library to reaching our audiences remotely.

**Conclusions:**

Our communication plan has provided the foundation for all of our messaging, be it print or digital media. In recent moments of crisis, it has been especially helpful for planning and executing large scale messaging. Similarly, knowing whom to contact around our organization to promote our message in different and broader ways has been extremely beneficial.

## BACKGROUND

How and when library staff communicates with library users reveals much about the relationship with and understanding of them. Developing effective strategies for reaching a variety of users is a challenging, iterative process that continues to evolve as technology and users' needs grow and change. But the unchanging truth is the indispensability of these strategies. According to Duhon and Jameson, “at its most basic level, the purpose of library outreach is universal: to reach as many patrons as possible in an effort to inform them about authoritative resources, which may be beyond their awareness or means to access” [1]. This is true regardless of the user librarians are trying to engage with, although they can usually be assigned to three larger categories: the physical library user, the online library user, and the library nonuser. Staff in medical or hospital libraries know reaching users can feel like an arduous task. Researchers, students, and clinicians have tight, overflowing schedules and high expectations when it comes to assistance.

So how can librarians communicate efficiently and effectively with these populations? How can they reach all three user categories in the most impactful ways? The key is through varied communication tactics and department partnerships. According to Swanberg et al., the latter is especially important: “One of the greatest values of these partnerships is leveraging partners' connections to the community in order to reach new audiences that may not have been participants otherwise” [2]. A partner, in most cases, equals an advocate. Developing relationships with advocates can help grow awareness of the library and utilizes a varied communication method to reach individuals who may otherwise be missed. It creates audience buy-in and trust when an outside party like a department representative recommends a library's services. Pozdol reinforces this notion, arguing that “to increase library involvement effectively and efficiently, it is helpful to determine which people in a school have the most influence or are best able to make things happen” [3]. Therefore, knowing your library advocates, your most effective communication methods, and your target audiences are all key to effectively spreading your message.

The Harvey Cushing/John Hay Whitney Medical Library, part of the Yale Library system, serves over 22,000 users primarily from the Yale Schools of Medicine, Public Health, and Nursing; the Physician Associate Program; the Online Physician Assistant Program; and the Yale New Haven Hospital (YNHH). The medical library is located in the Yale School of Medicine, centrally positioned on Yale's medical campus. The full-service library offers clinical, educational, and research support and services as well as access to a distinguished medical historical collection. On average, the library has 700 visitors each day.

Our communication plan was created in early 2019 by the medical library's Marketing and Communications Committee (supplemental Appendix A). The impetus for the plan's creation came from the committee's desire to deactivate the medical library's Facebook account. Initially, there were concerns about how we would reach our audiences if we no longer maintained a Facebook profile. To alleviate concerns, the committee created the communication plan to illustrate the myriad of ways that we communicated as well as explicitly identify the users we endeavored to reach.

The plan details our target audiences and segments them into primary, secondary, and tertiary categories. Each target audience group includes our goals for communication, methods for reaching these users, frequency of contact, and the library groups or individuals responsible for communicating with each. The communication plan follows the traditional format used in other industries; templates can be found through a simple web search for “communication plan.” Our communication plan complements our marketing strategy that outlines our goals for marketing and outreach during the upcoming year. In addition, we also maintain a list of public relations, communication, and library advocate contacts at Yale and beyond for our primary, secondary, and tertiary audiences.

In many ways, the plan merely captures and formalizes institutional knowledge of our audiences and established communication strategies. Though it was initially created in response to a concern, the utility of this plan has proved valuable in ways that we did not anticipate. For instance, we shared this document with the central library communication director to underscore the differences in target audiences between main campus libraries and the medical library. Being able to share this formalized plan reinforced our efforts to position our marketing and outreach initiatives with a medical community focus, instead of relying on central Yale Library initiatives that might not resonate with our users.

## CASE PRESENTATION

### Before building shutdown

Coronavirus disease 2019 (COVID-19), an infectious disease caused by a strain of coronavirus originating in China, quickly became a pandemic as it spread across the globe in early 2020 [4]. It entered the United States on January 15, 2020 [5]. By March, preparations for its imminent arrival in New Haven were underway across campus. Communication was essential during the week leading up to the first positive case at Yale, as many campus and library policies were updated daily, if not hourly. During this week, the medical library focused heavily on communicating virus prevention strategies and service changes to our users through website updates and signage in the library.

On March 10, Yale classes were moved online, and students were asked not to return to campus. On March 11, access to all libraries was restricted to Yale ID holders and expanded to include YNHH ID holders for the medical library.

Following these announcements, our immediate priority was to communicate the library's reduced hours of operation and new building access restrictions to users and potential visitors (mainly our primary and secondary audiences, and to a lesser extent, our tertiary audiences). To reach as many potential visitors as possible, our strategies were twofold: physical signage and website notices. We placed a large sign at the entrance of the medical library to inform users that access to the medical library was limited to Yale and hospital ID holders. We also added a notice to the top of the medical library website and created a “COVID-19 Library Update” blog post that detailed the changes to building access and provided an area to aggregate links to online resources like interlibrary loan, remote access, course reserves, and librarian consultations (supplemental Appendix B). This blog post became the central hub for all COVID-19 related library service updates and resources. Every time new content was added, the time stamp at the top of the page was updated.

Our next priority was to convey public health–focused guidelines for in-person visitors. Given the new access restrictions, visitors were limited to our primary audiences (i.e., users from the Yale medical center and YNHH). To encourage social distancing, hand washing, and other disease prevention methods, we printed large, informative posters and placed them near restrooms and high traffic areas. We also adjusted seating configurations in meeting rooms, classrooms, and study spaces in alignment with social distancing protocols (limiting conference rooms to three people instead of six, or table capacity to two instead of four), with signs explaining the new configurations.

Digital signage near the library entrance displayed rotating banners with messages like “wash your hands frequently for at least twenty seconds” to reinforce guidelines recommended by the Centers for Disease Control and Prevention to prevent the spread of COVID-19 [6]. To limit possible exposure, circulation staff members remained in an office behind the circulation desk, and signs were displayed asking users who needed assistance to ring the bell.

The effectiveness of these signs varied from space to space. In our silent reading rooms, users adhered to the new seating configurations as most were doing independent work. In our more collaborative spaces (like conference rooms or computer pods), we found users ignored the adjusted configurations and moved chairs back into these spaces or continued to sit in close proximity to one another. As COVID-19 was not “officially” in New Haven, users might not have felt an imminent threat.

On March 12, we moved all medical library workshops and office hours online. To signal this change, we added “Online Session” to the titles of each calendar event and emailed workshop registrants about the new format. As it was quickly becoming clear that a building closure was forthcoming, liaison librarians sent emails to faculty and instructors reinforcing that the medical library was ready to support them in online teaching.

The first positive case of COVID-19 at Yale was announced on Saturday, March 14, 2020 [7]. Shortly after, all Yale library buildings were closed until further notice.

### After building shutdown

Following the announcement of Yale's first COVID-19 case and the subsequent building closures, our priorities pivoted to communicating continuity of services and how to access remote resources.

The medical library's leadership team and communications coordinator had an emergency Zoom meeting immediately after the Saturday afternoon announcement to discuss next steps for communicating with our users. Our goals were to ensure that the community not only knew about the building's closure, but also about the plethora of online resources and services that library staff were still able to support remotely. After consulting the communication plan, we agreed the most effective way to communicate would be through an email. Using our Salesforce email platform, YaleMessage, we targeted 15,000 subscribers who had opted in to communications from the medical library, normally for quarterly updates on library news. This subscriber list enabled us to reach a large portion of our primary, secondary, and tertiary audiences.

On the afternoon of Sunday March 15, we sent an email to 15,329 subscribers (supplemental Appendix C). The message focused on how to get virtual support from library staff, how to set up remote access, how to access online collections, a list of available services and tools, and free COVID-19 related literature from publishers. Our intention was to reinforce our continued support while being as succinct as possible. The final word count was 501. Despite being sent on a Sunday, the email achieved a 47.8% open rate, with the majority of opens occurring within the first 24 hours.

Many medical students are unlikely to subscribe to the medical library's newsletter and, therefore, would not have received Sunday's email, so we crafted a separate email for medical students to remind them of the library's resources, of how to access them remotely, and that we were there to help. Additionally, we highlighted specific items we licensed like board review materials. We focused on brevity and included only the most important resources and information. The final word count was 340 (supplemental Appendix D). Because we were targeting a much smaller number of recipients, this email was sent from the students' personal librarians through Outlook, which also gave the email a more personalized touch.

Finally, to reach our primary audience of clinicians and users at YNHH, we partnered with colleagues at YNHH to distribute a clinical resource–focused email. Once again, we aimed to make the email as short as possible, including only key resources and tools. This email totaled just 204 words (supplemental Appendix E).

During this time, Yale Library's communication director was also planning to send an email to the Yale community regarding service continuity and remote resources at main campus libraries. Here again, we found unexpected utility in our communication plan. Many of our policies and procedures differed from those at the main campus libraries, so we made a case for segmented campus emails. Using our communication plan, we emphasized the differences between the target audiences for the medical library and the main campus libraries and demonstrated how and when we communicated with our community. The communication director agreed to leave the medical community off the main campus library emails, which in turn meant our audiences did not receive conflicting or redundant information.

## DISCUSSION

When faced with an unpredictable situation, we are best served by being nimble and innovative. Though we make every effort to be proactive and plan for a variety of scenarios, sometimes reacting is all we can do. In the case of COVID-19 and its impact on the Yale community, knowing our audience and how we could reach them was essential. Argenti et al. encourage carefully considering the objectives for each communication in order to understand what type of message to deliver (will it be one of utility? educational? promotional?) and then using the most effective channel to reach each specific audience [8].

Before the library was shut down, our immediate concern was communicating with users inside the building. We focused on physical and digital signage for in-person visitors, informing them about ways to prevent COVID-19 transmission as well as changes in library access. We updated our website frequently to inform online visitors about changes to policies and hours and began cultivating a list of remote resources for when the building was eventually closed. When the library was shut down, our focus shifted to reaching our audiences remotely. In both situations, we considered how our community already communicated and coordinated with our parent organization to ensure we reached our users through their preferred ways, a method detailed in Rossmann's article on strategic communications in libraries [9]. We used websites, library advocates, and the university's email platform to ensure we reached our users where they would be listening.

Though many of our initial communications were reactive (responding to the building closure and questions from users regarding services and collections), as time went on, they became more proactive. In each of these communication methods, we kept our message consistent and clear: though everyone is dealing with uncertainty, we are here to help. Argenti et al. reinforce that messages must all sound like they are coming from the same place with one clear direction [8]. We used a variety of channels to spread our announcements, but we remained consistent in tone and message.

Looking ahead, we are confronting the economic impacts of the virus on our budget in the fiscal years to come. Cuts to our collections budget and other spending will have ramifications across our medical campus and larger community, and communicating these decisions will be difficult. However, the same strategies will apply: a direct, brief, and supportive voice in the face of hardship. Effective strategic communication is based on truth [8]. Proactive communications about reductions are crucial to maintaining the trust we have developed with our community over time. Managing expectations from the start can help prevent frustrating discoveries later.

The key in all of these situations is knowing exactly how and whom to contact to make sure messages reach the people who need to hear them. We cannot overstate the value of a detailed communication plan and cultivating relationships with communications partners around an organization. Our communication plan has provided the foundation for all of our messaging, be it print or digital media. In these moments of crisis, it has been especially helpful for planning and executing large scale messaging. Similarly, knowing whom to contact around our organization to promote our messages in different and broader ways has been extremely beneficial. The medical library's communication plan coupled with communications connections in the larger Yale community were critical to ensuring this process was successful.

## Data Availability

There are no data associated with this article.

## SUPPLEMENTAL FILES

**Appendix A:** Harvey Cushing/John Hay Whitney Medical Library communication plan

**Appendix B:** Original “COVID-19 Library Update” blog post on the medical library website

**Appendix C:** Email from John Gallagher to the medical community, March 15, 2020

**Appendix D:** Email from personal librarian to medical students, March 17, 2020

**Appendix E:** Email to Yale New Haven Hospital (YNHH) community, March 20, 2020
