# Supplementary material for: Communicating with medical library users during COVID-19
Source: J Med Libr Assoc. 2021 Jan 1;109(1):107–11. doi: 10.5195/jmla.2021.1003 (PMC7772968; doi:10.5195/jmla.2021.1003)
Supplement: Supplementary file 1 — Appendix A: Harvey Cushing/John Hay Whitney Medical Library communication plan [file jmla-109-1-107-s01.pdf]

JMLA Journal of the Medical Library Association

Supplemental content to
J Med Libr Assoc. Jan;109(1):dx.doi.org/10.5195/jmla.2021.1003
www.jmla.mlanet.org
© Haugh 2021

Communicating with medical library users during COVID-19

Dana Haugh, MLS

APPENDIX A

Harvey Cushing/John Hay Whitney Medical Library communication plan

Primary audience

Audience	Goal	Format	Frequency	Owner
Yale Medical Center: includes Graduate Medical Education Community, which includes: residents, fellows, attendings, program directors, associate program directors, and program coordinators	• Build awareness of library classes, services, and resources<br>• Create desire to visit and utilize library services and tools<br>• Cultivate library-patron relationships<br>• Create partnerships among departments	Primary: Blog posts, digital signage, flyers, emails, web banners, Yale School of Medicine (YSM) digest, YSM calendar, tabling events, personal/department librarians, annual reports<br>Secondary: News coverage, campus presentations, event attendance	Varies (multiple times a month)	Liaison librarians; library staff; library director
Yale New Haven Hospital (YNHH)	• Build awareness of library classes, services, and resources<br>• Create desire to utilize library services and tools<br>• Encourage interaction between hospital and library	Primary: Blog posts, digital signage, flyers, emails, web banners, YNHH bulletin, tabling events, annual reports<br>Secondary: News coverage, campus presentations, event attendance	Varies (multiple times a month)	Clinical librarians; Marketing and Communications Committee; library director

JMLA Journal of the Medical Library Association

Supplemental content to
J Med Libr Assoc. Jan;109(1):dx.doi.org/10.5195/jmla.2021.1003
www.jmla.mlanet.org
© Haugh 2021

Secondary audience

Audience	Goal	Format	Frequency	Owner
Yale University Library staff	• Build awareness of library classes and services<br>• Encourage cross-library collaborations	Primary: Website/blog, emails, Yale University Library (YUL) calendar, Cushing/Whitney Medical Library (CWML) calendar, committee work, annual reports<br>Secondary: News coverage, presentations, events, trainings	As needed	Marketing and Communications Committee
Yale Colleges and Center for Science and Social Science Information (CSSSI)	• Build awareness of library classes, services, and resources<br>• Encourage use of collections<br>• Collaborations among campus departments	Primary: Website/blog, Yale calendar, YUL calendar<br>Secondary: News coverage	Monthly	Marketing and Communications Committee
West Campus Centers	• Awareness of library classes, services, and resources<br>• Encourage use of collections	Primary: Website/blog, newsletter, Yale calendar<br>Secondary: News coverage	Quarterly	Marketing and Communications Committee
Donors and associates	• Updates on library goings-on<br>• Funding opportunities<br>• Special events	Primary: Website/blog, targeted emails, annual reports<br>Secondary: News coverage	Few times a year	Library director; Marketing and Communications Committee

JMLA Journal of the Medical Library Association

SC
Supplemental content to
J Med Libr Assoc. Jan;109(1):dx.doi.org/10.5195/jmla.2021.1003
www.jmla.mlanet.org
© Haugh 2021

Tertiary audience

Audience	Goal	Format
Outside Yale (New England Public)	• Awareness of special initiatives<br>• Promotion of the Cushing Center	Primary: Website/blog, Yale calendar<br>Secondary: News coverage; Yale, YSM, YUL social media
Peer institutions	• Updates on library goings-on<br>• Awareness of special initiatives	Primary: Website/blog, email discussion lists, conference presentations, Yale calendar, professional organizations<br>Secondary: News coverage; Yale, YSM, YUL social media
Medical libraries; historical/archival collections	• Updates on special collections and acquisitions<br>• Awareness of research opportunities	Primary: Website/blog, email discussion lists, conference presentations, Yale calendar, professional organizations<br>Secondary: News coverage; Yale, YSM, YUL social media
Potential researchers	• Awareness of research opportunities<br>• Updates on special collections and acquisitions	Primary: Website/blog, email discussion lists, conference presentations, professional organizations<br>Secondary: News coverage; Yale, YSM, YUL social media
Hospital patients	• Awareness of library classes, services, and resources	Primary: Blog posts, web banners, YNHH bulletin, tabling events<br>Secondary: News coverage
