# Supplementary material for: Communicating with medical library users during COVID-19
Source: J Med Libr Assoc. 2021 Jan 1;109(1):107–11. doi: 10.5195/jmla.2021.1003 (PMC7772968; doi:10.5195/jmla.2021.1003)
Supplement: Supplementary file 2 — Appendix B: Original “COVID-19 Library Update” blog post on the medical library website [file jmla-109-1-107-s02.pdf]

JMLA Journal of the Medical Library Association

SC
Supplemental content to
J Med Libr Assoc. Jan;109(1):dx.doi.org/10.5195/jmla.2021.1003
www.jmla.mlanet.org
© Haugh 2021

Communicating with medical library users during COVID-19

Dana Haugh, MLS

APPENDIX B

Original "COVID-19 Library Update" blog post on the medical library website

COVID-19 Library Update

11 March 2020, 1:53 p.m. by Dana Haugh

Updated: 3/13/20 at 11:13 a.m.

Yale has instituted practices and policies to stem the spread of the global COVID-19 pandemic. The latest information may be found on the university's coronavirus page (https://communications.yale.edu/covid-19-information).

We appreciate your patience as we translate the university's latest guidelines into library procedures. Depending on staffing and as the situation evolves, library hours may be subject to change. As this unprecedented challenge evolves, we are actively working to develop service continuity plans that ensure a continued high level of support for our users.

Library Hours

Effective 3/13/20, medical library hours will be as follows:

Monday–Thursday: 8:00 a.m.–8:00 p.m.

Friday: 8:00 a.m.–6:00 p.m.

Saturday–Sunday: 10:00 a.m.–6:00 p.m.

- ☐ All Yale libraries remain open at this time, and we continue to provide services to students, faculty, and staff in the libraries as well as through our online platforms.
- ☐ Access to all Yale libraries is limited to holders of a valid Yale ID. Access to the Cushing/Whitney Medical Library will also be open to holders of Yale New Haven Hospital IDs.
- ☐ Our special collections reading rooms are also closed to visiting researchers until further notice, except for those who were already present and working in one of the repositories when this change went into effect.

Virtual Services

- ☐ Interlibrary Loan (https://library.medicine.yale.edu/services/ill)
- ☐ Course Reserves (https://library.medicine.yale.edu/services/course-reserves)
- ☐ Clinical and research support: Your departmental librarian is available via email and phone (https://library.medicine.yale.edu/research-help/find-your-librarian)

JMLA Journal of the Medical Library Association

SC
Supplemental content to
J Med Libr Assoc. Jan;109(1):dx.doi.org/10.5195/jmla.2021.1003
www.jmla.mlanet.org
© Haugh 2021

- ☐ Medical Student Support: Your personal librarian is available via email and phone (https://library.medicine.yale.edu/personal-librarian-lookup)
- ☐ Online Research Guides (https://library.medicine.yale.edu/guides)
- ☐ Tutorials (https://library.medicine.yale.edu/tutorials)
- ☐ Online library instruction (help with EndNote, PubMed, etc.) (https://library.medicine.yale.edu/classes)

Instruction Sessions and Tours

- ☐ The medical library's instruction sessions have moved online or have been cancelled. We encourage you to check the Classes page (https://library.medicine.yale.edu/classes) to stay updated on library sessions.
- ☐ All Cushing Center tours are cancelled until further notice.

Meeting rooms

- ☐ Meeting room reservations are currently suspended. If you have questions, please contact Victoria Helwig, 203.785.5352.
- ☐ Meeting room capacities have temporarily change in order accommodate recommended social distancing.

Recommendations from the Centers for Disease Control and Prevention (CDC)

- ☐ Stay at least six feet away from other people when possible
- ☐ Wash your hands frequently and for at least for twenty seconds
- ☐ Cover your mouth and nose when sneezing or coughing
- ☐ Stay home when you are sick
- ☐ Clean/disinfect frequently touched surfaces

We appreciate your patience as we translate the university's latest guidelines into library procedures. We are developing ways to conduct research and reference consultations by Zoom, and we are working to make as much of our physical course reserve materials available digitally as possible. Yale faculty, staff, and students should watch this space for further updates and check posted library hours (https://library.medicine.yale.edu/about/hours) immediately before visiting. Please direct any other questions to AskYaleMedicalLibrary@yale.edu.

Dana Haugh's blog (https://library.medicine.yale.edu/blog/8281)
