# Supplementary material for: Communicating with medical library users during COVID-19
Source: J Med Libr Assoc. 2021 Jan 1;109(1):107–11. doi: 10.5195/jmla.2021.1003 (PMC7772968; doi:10.5195/jmla.2021.1003)
Supplement: Supplementary file 3 — Appendix C: Email from John Gallagher to the medical community, March 15, 2020 [file jmla-109-1-107-s03.pdf]

JMLA Journal of the Medical Library Association

SC
Supplemental content to
J Med Libr Assoc. Jan;109(1):dx.doi.org/10.5195/jmla.2021.1003
www.jmla.mlanet.org
© Haugh 2021

Communicating with medical library users during COVID-19

Dana Haugh, MLS

APPENDIX C

Email from John Gallagher to the medical community, March 15, 2020

Dear community members,

For the safety of patrons and staff, the medical library and 24/7 room are closed until further notice. Library staff will continue to support you. Please visit our website for the most current information.

Virtual Support from Your Librarian

Please email your departmental librarian or personal librarian for support or to schedule a consultation by phone or Zoom.

For general questions, contact AskYaleMedicalLibrary@yale.edu. You can speak to a librarian on our virtual reference chat through our website. Research librarians are available daily from 8:00 a.m.–8:00 p.m.

Remote Access

Remote access to our vast electronic resources is available to the Yale and Yale New Haven Hospital (YNHH) community regardless of your location. For instructions on how to connect remotely, visit https://library.medicine.yale.edu/services/library-technology/remote.

Scanning Articles and Book Chapters

The library will continue to process interlibrary loan and scanning requests for articles and chapters. However, the fulfillment of these requests may be dependent on our partner libraries remaining open. Please prioritize requests that are the most urgent for your courses, research, policy decisions, and patient care. Submit interlibrary loan requests.

Books

At this time, checking out books, physical interlibrary loan of books, and BorrowDirect are suspended. Please keep any books you have in your possession; there is no need to return them at this time. All fines and fees for overdue items will be forgiven.

Online Classes, Tutorials, and Guides

- Instruction sessions and workshops continue online via Zoom.
- Research tutorials cover everything from how to manage citations to systematic searches to finding articles in PubMed.

Supplemental content to
J Med Libr Assoc. Jan;109(1):dx.doi.org/10.5195/jmla.2021.1003
www.jmla.mlanet.org
© Haugh 2021

- Research guides are subject- and department-specific collections of tools, databases, and resources aggregated by our medical librarians.
- Clinical/YNHH Resources lists tools for point-of-care, drug information, evidence-based practice, and more.
- Educational Software lists biomedical education resources.

Freely Accessible Literature on COVID-19

Many publishers are offering free access to literature on the COVID-19 pandemic:

- Springer Nature: SARS-CoV-2 and COVID-19
- Elsevier: Novel Coronavirus Information Center
- BMJ: Coronavirus (COVID-19): Latest News and Resources
- JAMA: Coronavirus Disease 2019 (COVID-19)
- Lancet: COVID-19 Free Resource Center
- NEJM: Coronavirus (COVID-19)
- UpToDate: Coronavirus Disease 2019 (COVID-19)
- Wiley: COVID-19: Novel Coronavirus outbreak
- Sage: Coronavirus related articles (PDF)
- DynaMed: COVID-19 (Novel Coronavirus)
- BioOne Complete: Peer-Reviewed Research to Inform the COVID-19 Crisis
- Johns Hopkins Dashboard: This dashboard leverages data from a number of national and global sources, and monitors the current scenario of COVID-19. Data are available in CSV format and available at the country, province, and date levels.
- GitHub containing the code behind the dashboard, as well as the World Health Organization (WHO) data behind these visualizations
- MIDAS 2019 Novel Coronavirus Repository: This repository serves as a central platform to share resources relevant for modeling of the COVID-19 outbreak.
- World Health Organization (WHO) Situation Reports: Access the latest situation updates and data regarding the COVID-19 outbreak provided by WHO.

Please know that the library is here to help you during this uncertain time.

John Gallagher, MLS
Director, Harvey Cushing/John Hay Whitney Medical Library, Yale School of Medicine
library.medicine.yale.edu
