# Supplementary material for: Communicating with medical library users during COVID-19
Source: J Med Libr Assoc. 2021 Jan 1;109(1):107–11. doi: 10.5195/jmla.2021.1003 (PMC7772968; doi:10.5195/jmla.2021.1003)
Supplement: Supplementary file 4 — Appendix D: Email from personal librarian to medical students, March 17, 2020 [file jmla-109-1-107-s04.pdf]

JMLA Journal of the Medical Library Association

SC
Supplemental content to
J Med Libr Assoc. Jan;109(1):dx.doi.org/10.5195/jmla.2021.1003
www.jmla.mlanet.org
© Haugh 2021

Communicating with medical library users during COVID-19

Dana Haugh, MLS

APPENDIX D

Email from personal librarian to medical students, March 17, 2020

Dear students,

Wild times, right?

For the safety of our staff and the extended Yale community, the Medical Library building and all Yale library buildings are now fully closed. Therefore, until further notice, the following services are suspended: check-out of library materials, physical interlibrary loan of books, and BorrowDirect. We cannot provide any access to library spaces or materials on library hold shelves at this time.

Nevertheless, we are here to help you remotely!

You can reach out to me with questions or to schedule an online meeting. We also have a chat feature up on the website if you need an immediate answer—a research librarian is monitoring it from 8:00 a.m.–8:00 p.m. every day.

Off-Campus Access to Library Resources

To access our resources remotely, you will need to download and install Yale's virtual private network (VPN) software and authenticate your sign-in with duo multifactor authentication (MFA). Here is a quick video tutorial to demonstrate how to sign in to the VPN.

Online Classes, Tutorials, and Guides

We may not be in the library, but our work hasn't stopped. We are offering workshops, consultations, and other relevant support. We are looking into licensing resources that will accommodate students' continuing education needs, especially board prep materials.

Don't see something you need? Email me, and we can find a way to make it work.

- Board prep on ExamMaster and clerkship study materials on AccessMedicine.
- Instruction sessions and workshops continue online via Zoom.
- Clinical/Yale New Haven Hospital (YNHH) resources lists tools for point-of-care, drug information, evidence-based practice, and more.
- Educational software lists biomedical education resources.
- Research tutorials cover everything from how to manage citations to systematic searches to finding articles in PubMed.
- Research guides are subject- and department-specific collections of tools, databases, and resources curated by our medical librarians.

JMLA Journal of the Medical Library Association
SC
Supplemental content to
J Med Libr Assoc. Jan;109(1):dx.doi.org/10.5195/jmla.2021.1003
www.jmla.mlanet.org
© Haugh 2021

Books, Interlibrary Loan, BorrowDirect, and more...

Please keep all physical items until the libraries reopen. All fines and late fees will be waived. Please don't mail materials back to us at this time.

As always, we are here for you, especially in these unusual and stressful times.

All the best, [Personal Librarian]
