# Supplementary material for: Communicating with medical library users during COVID-19
Source: J Med Libr Assoc. 2021 Jan 1;109(1):107–11. doi: 10.5195/jmla.2021.1003 (PMC7772968; doi:10.5195/jmla.2021.1003)
Supplement: Supplementary file 5 — Appendix E: Email to Yale New Haven Hospital (YNHH) community, March 20, 2020 [file jmla-109-1-107-s05.pdf]

JMLA Journal of the Medical Library Association

SC
Supplemental content to
J Med Libr Assoc. Jan;109(1):dx.doi.org/10.5195/jmla.2021.1003
www.jmla.mlanet.org
© Haugh 2021

Communicating with medical library users during COVID-19

Dana Haugh, MLS

APPENDIX E

Email to Yale New Haven Hospital (YNHH) community, March 20, 2020

Dear YNHH community,

Library staff are providing critical services remotely while the library building is closed. Please keep all books until the libraries reopen; all fines will be waived. We continue to provide free article delivery.

Contact us:

- Email clinical.librarians@yale.edu with clinical or research questions or to schedule an online consultation. We are prioritizing COVID-19-related research questions, but all inquiries will be answered quickly.
- Chat with a librarian if you need an immediate answer. Staff are monitoring questions from 8:00 a.m.–8:00 p.m. every day including weekends.

Other important resources available on the Medical Library website include:

- Remote support and resources from the library including a collection of COVID-19-related free literature repositories.
- Clinical/YNHH resources lists tools for point-of-care, drug information, evidence-based practice, and more. Many of these resources are easily available in EPIC under the “Library Services” tab.
- ClinicalKey and ClinicalKey Nursing offer procedural videos and clinical skill reviews.
- Library workshops continue online via Zoom.
- Research tutorials and research guides are available 24/7.

We appreciate your continued focus on patient care through these unusual and stressful times. As always, we are here to support you.

Please reach out with your questions.

John Gallagher, Director
Harvey Cushing/John Hay Whitney Medical Library
